# The antioxidant *N*-acetyl cysteine suppresses lidocaine-induced intracellular reactive oxygen species production and cell death in neuronal SH-SY5Y cells

**DOI:** 10.1186/s12871-016-0273-3

**Published:** 2016-10-24

**Authors:** Akihisa Okamoto, Masahiro Tanaka, Chisato Sumi, Kanako Oku, Munenori Kusunoki, Kenichiro Nishi, Yoshiyuki Matsuo, Keizo Takenaga, Koh Shingu, Kiichi Hirota

**Affiliations:** 1 Department of Anesthesiology, Kansai Medical University, Hirakata, Japan; 2 Department of Life Science, Shimane University School of Medicine, Izumo, Japan

**Keywords:** Lidocaine, Mitochondria, ROS, Redox, Apoptosis, Necrosis, Oxygen consumption

## Abstract

**Background:**

The local anesthetic lidocaine can affect intra- and extra-cellular signaling pathways in both neuronal and non-neuronal cells, resulting in long-term modulation of biological functions, including cell growth and death. Indeed, lidocaine was shown to induce necrosis and apoptosis in vitro. While several studies have suggested that lidocaine-induced apoptosis is mitochondrial pathway-dependent, it remains unclear whether reactive oxygen species (ROS) are involved in this process and whether the observed cell death can be prevented by antioxidant treatment.

**Methods:**

The effects of lidocaine and antioxidants on cell viability and death were evaluated using SH-SY5Y cells, HeLa cells, and HeLa cell derivatives. Cell viability was examined via MTS/PES ([3-(4,5-dimethylthiazol-2-yl)-5-(3-carboxymethoxyphenyl)-2-(4-sulfophenyl)-2H-tetrazolium, inner salt]/phenazine ethosulfate) assay. Meanwhile, cell apoptosis and necrosis were evaluated using a cell death detection assay with Annexin V-FITC and PI staining, as well as by assaying for caspase-3/7 and caspase-9 activity, and by measuring the release of lactate dehydrogenase, respectively. Mitochondrial transmembrane potential (ΔΨm) was assessed using the fluorescent probe tetramethylrhodamine ethyl ester.

**Results:**

Lidocaine treatment resulted in suppression of the mitochondrial electron transport chain and subsequent attenuation of mitochondrial membrane potential, as well as enhanced ROS production, activation of caspase-3/7 and caspase-9, and induction of apoptosis and necrosis in SH-SY5Y cells in a dose- and time-dependent manner. Likewise, the anesthetics mepivacaine and bupivacaine also induced apoptosis in SH-SY5Y cells. Notably, the antioxidants *N*-acetyl cysteine (NAC) and Trolox successfully scavenged the mitochondria-derived ROS and suppressed local lidocaine-induced cell death.

**Conclusions:**

Our findings demonstrate that the local anesthetics lidocaine, mepivacaine, and bupivacaine inhibited the activity of mitochondria and induced apoptosis and necrosis in a dose-dependent manner. Furthermore, they demonstrate that treatment with the antioxidants NAC, Trolox, and GGA resulted in preservation of mitochondrial voltage and inhibition of apoptosis via suppression of caspase activation.

**Electronic supplementary material:**

The online version of this article (doi:10.1186/s12871-016-0273-3) contains supplementary material, which is available to authorized users.

## Background

There is an established consensus that local anesthetics exert nerve-blocking activity, primarily through the inhibition of voltage-gated sodium channels [1]. However, these compounds can also affect the intra- and extra-cellular signaling pathways of both neuronal and non-neuronal cells, resulting in long-term modulation of biological functions, including cell growth and death [2]. Lidocaine is a widely used local anesthetic and anti-arrhythmic agent. Notably, lidocaine was also shown to induce apoptosis and necrosis both in vitro [3–5] and in vivo [6], and to cause transient or permanent nerve injury, such as *cauda equina* syndrome, after spinal anesthesia in clinical settings [7, 8]. In addition, it was reported that lidocaine inhibits the invasive ability of cancer cells at concentrations used for surgical operations (5–20 mM).

Apoptosis is a form of programmed cell death that is characterized by a series of distinct morphological and biochemical changes, and is an important process in a wide variety of biological systems. There are two major signaling pathways by which apoptosis is induced: the intrinsic and extrinsic pathway. While the extrinsic pathway is dependent on cell-surface death receptors such as Fas (First apoptosis signal), the intrinsic pathway is initiated within mitochondria [9]. Specifically, in the intrinsic pathway, the formation of a multimeric Apaf-1/cytochrome *c* complex results in activation of caspase-9, which in turn cleaves and activates the downstream caspases caspase-3, −6, and −7 [1, 10]. Notably, reactive oxygen species (ROS) are widely believed to play an essential role in apoptosis. Indeed, several studies indicate that ROS scavengers, including the synthetic compound *N*-acetyl cysteine (NAC) and the endogenous redox-active molecule thioredoxin (TRX), can be used to alleviate intracellular ROS and thereby prevent apoptosis and necrosis.

Previous studies demonstrated that lidocaine-induced cell death is dependent on the mitochondrial pathway; however, it is still largely unclear whether ROS are involved in this process. In this study, we demonstrate that in vitro lidocaine treatment resulted in attenuation of mitochondrial membrane potential and promoted caspase-dependent apoptosis in neuronal SH-SY5Y cells. Moreover, we show that the observed increases in cell death were mitochondria-derived ROS-dependent and could be blocked by treatment with several antioxidant compounds.

## Methods

### Reagents

Lidocaine, mepivacaine, bupivacaine, NAC, and (±)-6-Hydroxy-2,5,7,8-tetramethylchromane-2-carboxylic acid (Trolox) were obtained from Sigma-Aldrich (St. Louis, MO, USA), teprenone (geranylgeranylacetone, GGA) was obtained from Wako Pure Chemical Industries (Osaka, Japan), and recombinant human TRX (rhTRX) was obtained from Oriental Yeast Co., Ltd. (Tokyo, Japan). GGA and Trolox were dissolved in absolute ethanol, while bupivacaine and NAC were dissolved in H_2_O, and rhTRX was dissolved in citric acid. Rotenone, oligomycin and antimycin A were obtained from Abcam, Inc. (Cambridge, MA, USA).

### Cell culture

All cell lines were obtained from American Type Culture Collection (ATCC; Manassas, VA, USA). The established cell lines derived from human neuroblastoma SH-SY5Y cells and cervical carcinoma HeLa cells were maintained in Roswell Park Memorial Institute (RPMI) 1640 medium supplemented with 10 % fetal bovine serum, 100 units/ml penicillin, and 0.1 mg/ml streptomycin. The characteristics of EB8 cells (HeLa cells lacking mtDNA) and HeEB1 cells (a hybrid clone of EB8 cells containing mtDNA from wild-type HeLa cells) have been described elsewhere [11, 12]. All cells were maintained at 37 °C in a humidified atmosphere containing 5 % CO_2_. Cells were grown in 100 mm dishes and were subcultured for experiments when they reached 85 % confluence.

### Cell viability assay (MTS assay)

Cell viability was assessed using a CellTiter 96™ AQueous One Solution Cell Proliferation Assay (Promega, Madison, WI, USA). Briefly, SH-SY5Y cells were seeded into 96-well plates (2 × 10^4^ cells/well) and cultivated overnight. The following day, cells were treated with the indicated concentrations of the appropriate drug(s) for varying lengths of time. After treatment, 20 μl of CellTiter 96 AQueous One Solution™ Reagent was added to each well, the plates were incubated at 37 °C for 1 h, and the absorbance of each sample was measured using an iMark™ Micropate Reader (BIO-RAD, Hercules, CA, USA) at a wavelength of 490 nm. Cell viability was then calculated by comparing the absorbance of treated cells with that of the control cells (incubated without drugs), which was defined as 100 % [13, 14]. All samples were tested in triplicate for each experiment.

### Caspase-3/7 and caspase-9 activity assays

The levels of caspase-3/7 and caspase-9 activity were assessed using an Apo-ONE™ Homogeneous Caspase-3/7 Assay Kit (Promega) and a Caspase-Glo™ 9 Assays Kit (Promega), respectively, according to the manufacturer’s protocols. Briefly, SH-SY5Y cells were seeded into 96-well plates (2 × 10^4^ cells/well) and incubated overnight. The following day, cells were treated with the indicated concentrations of the appropriate drug(s) for varying lengths of time. After treatment, 100 μl of Apo-ONE Caspase-3/7 Reagent™ or Caspase-Glo 9 reagent™ was added to each well, respectively. Cells were incubated at room temperature for 1 h and the luminescence of each well was measured using an EnSpire™ Multimode Plate Reader (PerkinElmer, Waltham, MA, USA). Caspase activity was then calculated by comparing the levels of luminescence of the treated cells with that of the control cell population (incubated without drugs), which was defined as 100 %. Assays were performed in triplicate at least twice. Data were expressed as means ± standard deviations (SD).

### Immunoblot assays

Whole cell lysates were prepared as described previously [15, 16]. Briefly, cells were lysed by suspension in ice-cold lysis buffer [0.1 % sodium dodecyl sulfate (SDS), 1 % NP40, 5 mM ethylene diamine tetraacetic acid (EDTA), 150 mM NaCl, 50 mM Tris-Cl (pH 8.0), 1 mM sodium orthovanadate, and Complete Protease Inhibitor™ (Roche Applied Science)] and centrifuged at 10,000 × *g* to pellet cell debris. Approximately 25 μg of each protein sample was then separated by SDS-polyacrylamide gel electrophoresis (SDS-PAGE) and subjected to immunoblot analysis using rabbit polyclonal antibodies specific to PARP [poly (ADP-ribose) polymerase; 1:1,000] or cleaved caspase-9 (Asp315; 1:1,000) (Cell Signaling Technology, Danvers, MA, USA) [17], β-actin (Sigma-Aldrich), and anti-rabbit IgG horseradish peroxidase-linked secondary antibodies (1:2000 dilution; Cell Signaling Technology). Immunolabeled proteins were then visualized using enhanced chemiluminescence (ECL™) reagents (Amersham Biosciences, Little Chalfont, UK).

### Analysis of cell apoptosis

Levels of cell apoptosis were measured using an Annexin V-FITC Apoptosis Detection Kit™ (BioVision, Milpitas, CA, USA), according to the manufacturer’s instructions. For these analyses, SH-SY5Y cells were seeded into 6-well plates (3 × 10^5^ cells/well) and incubated overnight. The following day, cells were treated with the indicated concentrations of the appropriate drug(s) for varying lengths of time and harvested by centrifugation at 1200 rpm for 3 min. The culture supernatants were discharged, and the resulting pellets were suspended in a mixture comprised of 500 μl binding buffer, 5 μl Annexing V-FITC, and 5 μl propidium iodide (PI; 50 μg/ml) for 5 min at room temperature in the dark and analyzed using a FACSCalibur flow cytometer (BD Biosciences, San Jose, CA, USA) equipped with CellQuest Pro™ software [4, 13]. Data were evaluated using FlowJo™ version 7.6.3 software (TreeStar, Ashland, OR, USA), exported to Excel spreadsheets, and subsequently analyzed using the statistical application R.

### Lactate dehydrogenase (LDH)-based cytotoxic assay

Levels of cell cytotoxicity were evaluated using a CytoTox-ONE™ Kit (Promega). Briefly, SH-SY5Y cells were seeded into 96-well plates (2 × 10^4^ cells/well) and incubated overnight. The following day, cells were treated with the indicated concentrations of the appropriate drug(s) for varying lengths of time. Twenty microliters of CytoTox-ONE™ reagent was added to each well, plates were incubated at 22 °C for 10 min, and then 50 μl of Stop Solution was added to each well. The resulting fluorescence was measured using an EnSpire™ Multimode Plate Reader (PerkinElmer) at an excitation wavelength of 560 nm and an emission wavelength of 590 nm. Percentages of cell death were calculated by comparing the level of LDH released (fluorescence value) from each treatment group with that of the positive control population (cells treated with Lysis solution), which was defined as 100 %. Meanwhile, the level of LDH released from the negative control population (untreated cells) was defined as 0 %. All samples were evaluated in triplicate for each experiment.

### Determination of mitochondrial membrane potential (ΔΨm)

Mitochondrial membrane potential was determined by flow cytometry using a MitoPT™ JC-1 Assay Kit (ImmunoChemistry Technologies, Bloomington, MN, USA), according to the manufacturer’s instructions. For these analyses, SH-SY5Y cells were seeded into 6-well plates (3 × 10^5^ cells/well) and cultivated overnight. The following day, cells were treated with the indicated concentrations of the appropriate drug(s) for varying lengths of time and then pelleted by centrifugation at 1200 rpm for 3 min. Supernatants were discharged, and cells were resuspended in JC-1, incubated at 37 °C for 15 min in the dark, and collected by centrifugation at 1200 rpm for 3 min. Supernatants were again discharged and the remaining cell residues were suspended in 500 μl assay buffer. Samples were subsequently analyzed using a FACSCalibur flow cytometer (BD Biosciences, San Jose, CA, USA) equipped with CellQuest Pro™ software [4, 13] for the detection of red JC-1 aggregates (590 nm emission) or green JC-1 monomers (527 nm emission). The resulting data were evaluated using FlowJo version 7.6.3 software (TreeStar, San Carlos, CA), exported to Excel spreadsheets, and subsequently analyzed using the statistical application R.

### Measurement of total cellular O_2_ consumption rate (OCR)

Total OCR was measured as described previously [12, 16]. Briefly, SH-SY5Y cells were trypsinized and suspended at a concentration of 1 × 10^7^ cells/ml in RPMI containing 10 % FBS and 25 mM HEPES buffer. For each experiment, equal numbers of cells (suspended in 1 ml) were pipetted into the chamber of an Oxytherm electrode unit (Hansatech Instruments, Norfolk, UK), which uses a Clark-type electrode to monitor the concentration of dissolved O_2_ in the sealed chamber over time. The test reagents including lidocaine, rotenone and FCCP were added into the chambers immediately before each measurement. The resulting data were exported to a computerized chart recorder (Oxygraph; Hansatech Instruments), which calculated the OCR values. The temperature was maintained at 25 °C during measurement. The concentrations of O_2_ in 1 ml of DMEM medium lacking cells was also measured over time and utilized as the background. O_2_ consumption experiments were repeated at least three times, and data were expressed as means ± SD [12].

### Live cell ROS imaging

For evaluation of intracellular ROS generation, control and lidocaine-treated SH-SY5Y cells were treated with the ROS-sensitive dye 2', 7'-dichlorodihydrofluorescin diacetate (DCFH-DA) and analyzed using a BioStation IM live cell time-lapse imaging system (Nikon, Tokyo, Japan) at 37 °C and 5 % CO_2_; phase-contrast and fluorescence images were acquired at 15 min intervals [18, 19].

### Statistical analysis

All experiments were repeated at least twice and each sample was evaluated in triplicate. Representative data, expressed as means ± SD, are shown. Differences between results were evaluated by one-way analysis of variance (ANOVA) or two-way ANOVA, followed by Dunnett’s test for multiple comparisons. All statistical analyses were performed using EZR (Saitama Medical Center, Jichi Medical University), which is a graphical user interface for R (The R Foundation for Statistical Computing, version 3.1.3) [20]. More precisely, it is a modified version of R commander software (version 1.6–3) and includes statistical functions that are frequently used in biostatistics. *P*-values < 0.05 were considered statistically significant. All graphs were generated by the application Prism 6 for Mac OS X (GraphPad Software Inc., La Jolla, CA USA).

## Results

### Lidocaine induces SH-SY5Y cell death in a dose- and time-dependent manner

**Fig. 1:**
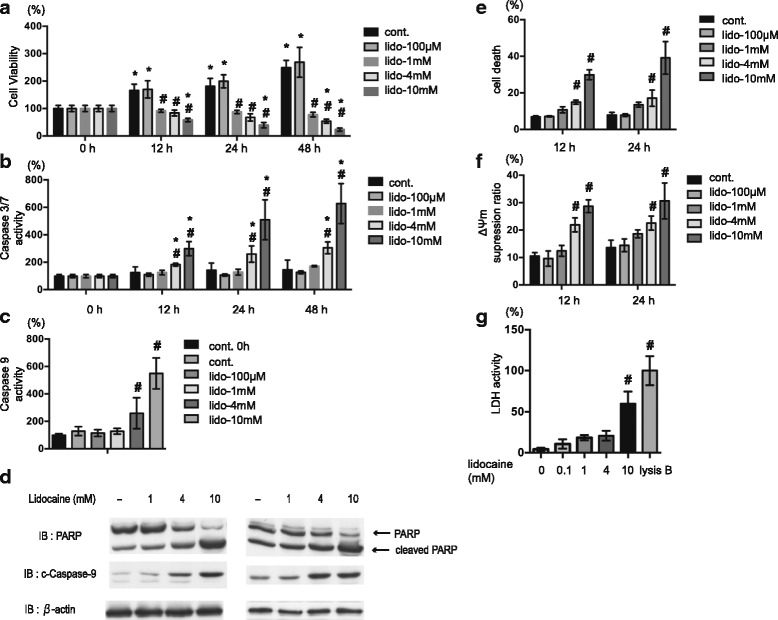
Lidocaine induces SH-SY5Y cell death in a dose- and time-dependent manner. Neuronal SH-SY5Y cells were exposed to the indicated concentrations (0.1, 1, 4, or 10 mM) of lidocaine for varying lengths of time (0, 12, 24, and 48 h). **a** Graphic depiction of the levels of cell viability of treated and untreated cells at each time point, as evaluated by MTS [3-(4,5-dimethylthiazol-2-yl)-5-(3-carboxymethoxyphenyl)-2-(4-sulfophenyl)-2H-tetrazolium] assay analysis (*n* = 4). **b** and **c** Graphic depictions of caspase-3/7 (*n* = 3) and caspase-9 (*n* = 5) activity in each treatment group at different time points, as determined using an Apo-ONE™ Homogeneous Caspase-3/7 Assay Kit and a Caspase-Glo™ 9 Assays Kit, respectively. **d** Immunoblot analysis of the levels of poly (ADP-ribose) polymerase (PARP), cleaved caspase-9 and β-actin in the lysates of treated and untreated cells after 24 h. The blots are derived from two independent experiments. **e** Treated and untreated cells were harvested, and the levels of cell death were analyzed by flow cytometry. The ratio of propidium iodide (PI)-positive and/or annexin V-positive cells [(Q1 + Q2 + Q4)/(Q1 + Q2 + Q3 + Q4)] was used to calculate the percentage of dead cells (Additional file 1: Figure S1) (*n* = 3). **f** Graphic depiction of the average mitochondrial membrane potential (ΔΨm) of treated and untreated cells (*n* = 3) at each time point, as measured using a MitoPT™ JC-1 Assay Kit. Values indicate the ratio [Q2/(Q2 + Q4)] of green JC-1 monomers (527 nm emission) to red aggregates (590 nm emission). **g** Graphic depiction of the levels of cell death among treated and untreated cell populations. Cell death was evaluated by measuring the levels of lactate dehydrogenase (LDH) within culture supernatants (*n* = 4). Control was treated by the lysis buffer. Data presented in **a**–**c** and **e**–**g** are expressed as means ± standard deviations (SD). Differences between results were evaluated by two-way ANOVA (**a**, **b**, **e** and **f**), followed by Dunnett’s test for multiple comparisons in each group and one-way ANOVA (**c** and **g**) followed by Dunnett’s test for multiple comparisons. *p < 0.05 compared with the control cell population at incubation time 0 h (no treatment). #*p* < 0.05 compared with the control cell population at the same time period (group)

MTS/PES ([3-(4,5-dimethylthiazol-2-yl)-5-(3-carboxymethoxyphenyl)-2-(4-sulfophenyl)-2H-tetrazolium, inner salt]/phenazine ethosulfate) assays were utilized to investigate the effects of lidocaine on neuronal cell proliferation or viability. In this assay, the NADPH and NADH produced by dehydrogenase enzymes of metabolically active cells bioreduce the MTS tetrazolium compound (Owen’s reagent) into a colored formazan product that is soluble in tissue culture medium. Treatment with 1 mM, 4 mM, and 10 mM lidocaine significantly suppressed SH-SY5Y cell viability at 12 h, 24 h, and 48 h after administration in both a dose- and time-dependent manner (Fig. 1a). In contrast, 100 μM lidocaine had no effect on cell viability (Fig. 1a). To elucidate the mechanism by which lidocaine suppresses cell viability, we evaluated the effect of this compound on the levels of apoptosis in SH-SY5Y cells. Notably, treatment with 4 mM or 10 mM lidocaine resulted in significant increases in caspase-3/7 activation after 12 h, 24 h, and 48 h (Fig. 1b), while treatment with 4 mM and10 mM lidocaine yielded statistically significant activation of caspase-9 (Fig. 1c), as well as increased levels of cleaved PARP and caspase-9 (Fig. 1d), after 24 h.

To confirm these findings, SH-SY5Y cells were stained with PI and FITC-conjugated annexin V and evaluated by flow cytometry. Cells treated with 4 mM or 10 mM lidocaine exhibited significantly increased numbers of PI-positive or annexin V-positive cells after 12 h and 24 h of treatment (Fig. 1e and Additional file 1: Figure S1A–S1E). Additionally, treatment with 4 mM or 10 mM lidocaine resulted in significant reductions in mitochondrial voltage (Fig. 1f and Additional file 2: Figure S2A–S2D), while treatment with 10 mM lidocaine resulted in significant increases in SH-SY5Y cell death after 12 h, as indicated by the levels of LDH release (Fig. 1g).

### Critical involvement of mitochondria in lidocaine-induced cell death

**Fig. 2:**
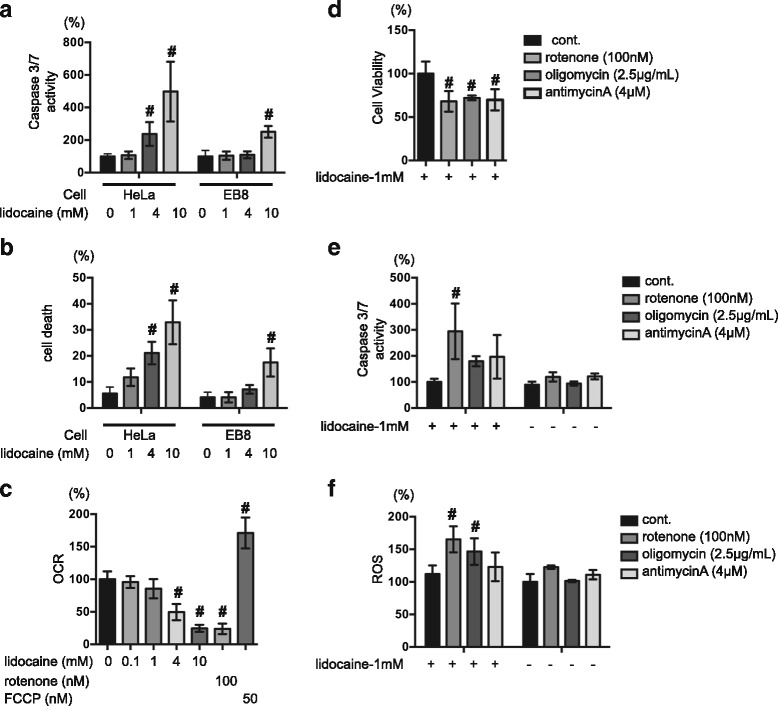
Critical involvement of mitochondria in lidocaine-induced cell death. HeLa cells and EB8 cells (HeLa cells lacking mitochondrial DNA) were exposed to the indicated concentrations of lidocaine (0, 0.1, 1, 4, or 10 mM) for 24 h. **a** Graphic depiction of the levels of caspase-3/7 activity in each treatment group, as determined using an Apo-ONE Homogeneous Caspase-3/7 Assay Kit™ (*n* = 4). **b** Treated and untreated cells were harvested, and the levels of cell death were analyzed by flow cytometry (*n* = 4). The ratio with propidium iodide (PI)- or annexin V-positive cells [(Q1 + Q2 + Q4)/(Q1 + Q2 + Q3 + Q4)] were indicated as dead cells (Additional file 1: Figure S1). **c** Graphic depiction of the oxygen consumption rate (OCR) in untreated SH-SY5Y cells and cells treated with lidocaine (0.1, 1, 4, or 10 mM), rotenone (100 nM), or carbonyl cyanide-p-trifluoromethoxyphenylhydrazone (FCCP) (50 nM). Values are presented as ratios of OCR compared to that in the control (without lidocaine treatment) group (*n* = 4). **d** and **e** Graphic depiction of the levels of cell viability among SH-SY5Y cells treated with mitochondrial ETC inhibitors. Cells were treated with 1 mM lidocaine and either 100 nM rotenone, 2.5 μg/ml oligomycin, or 4 μM antimycin A, and subjected to (**d**) MTS [3-(4,5-dimethylthiazol-2-yl)-5-(3-carboxymethoxyphenyl)-2-(4-sulfophenyl)-2H-tetrazolium] assay (*n* = 3) or (**e**) caspase-3/7 activity assay (*n* = 3) analysis. **f** Graphic depiction of reactive oxygen species (ROS) production in SH-SY5Y cells exposed to 1 mM lidocaine in the presence or absence of 100 nM rotenone, 2.5 μg/ml oligomycin and 4 μM antimycin A for 6 h (*n* = 3). Data depict the ratio of ROS production in treated cells compared to that in the untreated control group. All data were expressed as means ± standard deviations (SD). Differences between results were evaluated by two-way analysis of variance (ANOVA) (**a**, **b**, **e** and **f**) followed by Dunnett’s test for multiple comparisons in each group or one-way ANOVA (**c** and **d**), followed by Dunnett’s test for multiple comparisons. #*p* < 0.05 compared with the control treatment population in the same group

We next examined the effects of lidocaine treatment on HeLa cervical carcinoma cells, as well as the HeLa-derived cell lines EB8, which lack mitochondrial DNA (ρ0 cells), and HeEB1, a hybrid clone of EB8 cells containing mtDNA from wild-type HeLa cells. Compared to HeLa cells and HeEB1, EB8 cells exhibited lower levels of O_2_ consumption (Additional file 3: Figure S3A). Meanwhile, treatment with 4 mM and 10 mM lidocaine resulted in significant increases in caspase-3/7 activation and cell death in both HeLa cells (Fig. 2a) and HeEB1 cells after 24 h, compared to the control population (Additional file 3: Figure S3B and S3C). Conversely, only 10 mM lidocaine treatment induced caspase-3/7 activation in the EB8 ρ0 cell line (Fig. 2a). Notably, however, treatment with 10 mM lidocaine resulted in significantly higher levels of caspase-3/7 activity in HeLa cells than in EB8 cells. Moreover, flow cytometry analysis detected no significant difference in the levels of cell death between the untreated EB8 ρ0 control population and the population subjected to 4 mM-lidocaine treatment, indicating that, in contrast to the parental HeLa cell line, EB-8 cells are resistant to low levels of lidocaine (Fig. 2b). These results prompted us to examine the involvement of mitochondria in lidocaine-induced cell death. First, we investigated the effect of lidocaine on oxygen consumption in SH-SY5Y cells; compared with the untreated control population, SH-SY5Y cells treated with 4 mM and 10 mM lidocaine exhibited reduced OCRs (Fig. 2c). We next evaluated the effects of mitochondrial ETC. inhibitors, including 100 nM rotenone, 2.5 μg/ml oligomycin, and 4 μM antimycin A, on SH-SY5Y cells. As expected, each of these three reagents significantly suppressed MTS conversion (Fig. 2d). Treatment with 1 mM lidocaine or each ETC. inhibitors did not elicit caspase 3/7 activation or ROS generation in SH-SY5Y cells (Fig. 2e and f). Notably co-treatment with 1 mM lidocaine and rotenone effectively induced the caspase activation. (Fig. 2e and f). In this study, we used three types of ETC inhibitors. Although all the ETC inhibitors increased caspase 3/7 activity and ROS generation, only rotenone exerted statistically significant effect. Rotenone is a Complex I inhibitor. Oligomycin is an ATP synthase inhibitor. Antimycin A is a Complex III inhibitor. The evidence warrants further investigation to clearly elucidate the target(s) of lidocaine and the mechanism of the synergistic effect of lidocaine and ETC inhibitors.

These findings are therefore consistent with those obtained by flow cytometry and indicate that lidocaine promotes cell death by targeting mitochondria.

### Effect of synthetic antioxidants on lidocaine-induced cell death

**Fig. 3:**
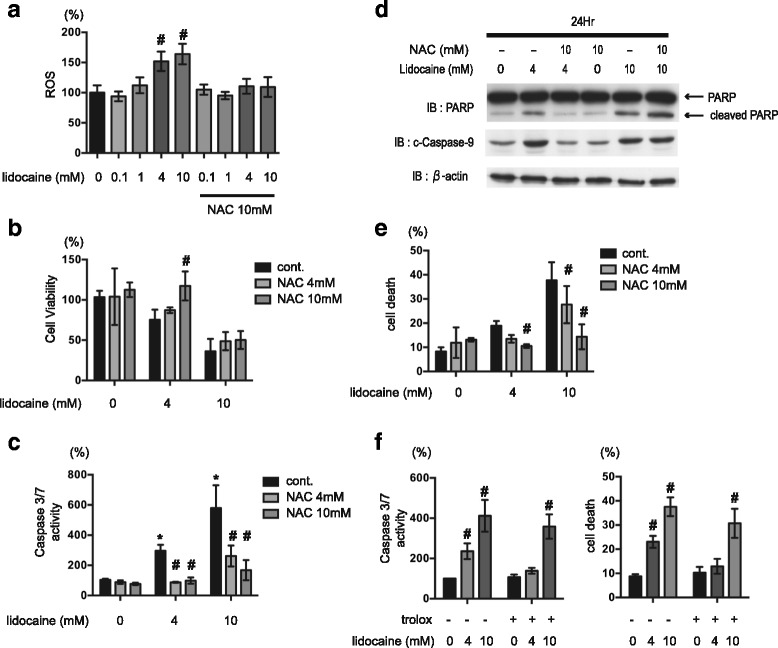
Effect of synthetic antioxidants on lidocaine-induced cell death. **a** Graphic depiction of reactive oxygen species (ROS) production in SH-SY5Y cells exposed to the indicated concentrations of lidocaine (0, 0.1, 4, or 10 mM) for 6 h (*n* = 3) in the presence or absence of 10 mM *N*-acetyl cysteine (NAC). Data depict the ratio of ROS production in treated cells compared to that in the untreated control group. **b**–**e** SH-SY5Y cells were exposed to the indicated concentrations of lidocaine (0, 4, or 10 mM) for 24 h in the presence or absence of NAC (4 or 10 mM). **b** Cell viability and (**c**) caspase-3/7 activity were evaluated by MTS [3-(4,5-dimethylthiazol-2-yl)-5-(3-carboxymethoxyphenyl)-2-(4-sulfophenyl)-2H-tetrazolium] assay (*n* = 3) and Apo-ONE™ Homogeneous Caspase-3/7 Assay (*n* = 3) analysis, respectively. **d** Cells were harvested and lysates were subjected to immunoblot assay analysis using antibodies specific to poly (ADP-ribose) polymerase (PARP) and cleaved caspase-9. **e** Graphic depiction of the levels of cell death among treated and untreated cell populations, as evaluated by flow cytometry (*n* = 4). The ratio with PI or annexin V positive cells [(Q1 + Q2 + Q4)/(Q1 + Q2 + Q3 + Q4)] were indicated as dead cells (Additional file 1: Figure S1). **f** SH-SY5Y cells were exposed to the indicated concentrations of lidocaine (0.1, 1, 4, or 10 mM) in the presence or absence of 250 μM Trolox for 24 h, and subjected to caspase-3/7 activity assay analysis (*n* = 4) (left panel). Graphic depiction of the levels of cell death among treated and untreated cell populations, as evaluated by flow cytometry (*n* = 3) (right panel). The ratio with PI or annexin V positive cells [(Q1 + Q2 + Q4)/(Q1 + Q2 + Q3 + Q4)] were indicated as dead cells. Differences between results were evaluated by one-way analysis of variance (ANOVA) (**a**) followed by Dunnett’s test for multiple comparisons or two-way ANOVA (**b**, **c**, **e** and **f**) followed by Dunnett’s test for multiple comparisons in each group. *p < 0.05 compared with the control cell population at 0 h (no treatment). #*p* < 0.05 compared with the control treatment population in the same group

Several reports have indicated that ROS play a critical role in mitochondria-dependent cell death. As demonstrated in Fig. 3a, 4 mM and 10 mM lidocaine treatment induced ROS accumulation in SH-SY5Y cells within 6 h. Notably, however, these increases in ROS were blocked upon treatment with the antioxidant NAC (Fig. 3a). We therefore further examined the effects of the antioxidant NAC on SH-SY5Y cells. Treatment with 10 mM NAC blocked the suppression of SH-SY5Y viability observed upon treatment with 4 mM lidocaine (Fig. 3b). Moreover, NAC inhibited the 4 mM lidocaine-induced increase in caspase-3/7 activity (Fig. 3c) in a dose-dependent manner. While neither 4 mM nor 10 mM NAC treatment had any significant effect on the suppression of cell viability mediated by 10 mM lidocaine treatment (Fig. 3b), the levels of caspase-3/7 activation induced by 10 mM lidocaine were partially suppressed by exposure to 4 mM and 10 mM NAC (Fig. 3c). Similar effects were observed in SH-SY5Y cells treated with another antioxidant, Trolox (250 μM; Fig. 3f). 250 μM Trolox suppressed ROS generation elicited by 4 mM lidocaine (Additional file 3: Figure S3E). 250 μM Trolox treatment significantly suppressed 4 mM-lidocaine induced cell death but not 10 mM-lidocaine induced death (Fig. 3f, right panel). Although neither 4 mM nor 10 mM-lidocaine induced caspase 3/7 activation was not statistically significant, 250 μM Trolox suppressed 4 mM-lidocaine induced the caspase activation. Likewise, flow cytometry analyses demonstrated the NAC treatment suppressed the effects of 4 mM and 10 mM lidocaine on cell death (Fig. 3e and Additional file 1: Figure S1F–S1H) and mitochondrial voltage (Additional file 2: Figure S2E–S2G). LDH release by 10 mM lidocaine was not suppressed by NAC or Trolox treatment (Additional file 3: Figure S3E).

### Effects of an endogenous antioxidant on lidocaine-induced cell death

**Fig. 4:**
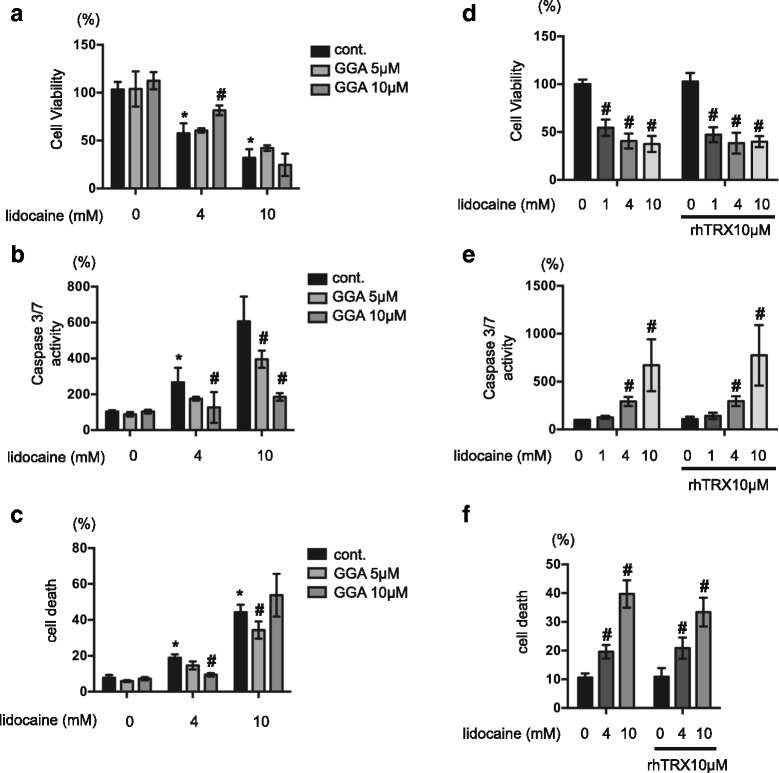
Effects of an endogenous antioxidant on lidocaine-induced cell death. **a**–**c** SH-SY5Y cells were exposed to the indicated concentrations of lidocaine (0, 4, or 10 mM) for 24 h in the presence or absence of pretreatment with 5 or 10 μM teprenone (geranylgeranylacetone, GGA) for 24 h. **a** Cell viability and **b** caspase-3/7 activity were evaluated by MTS [3-(4,5-dimethylthiazol-2-yl)-5-(3-carboxymethoxyphenyl)-2-(4-sulfophenyl)-2H-tetrazolium] and Apo-ONE™ Homogeneous Caspase-3/7 Assay analysis, respectively (*n* = 3 for each). **c** Graphic depiction of the levels of cell death among treated and untreated cell populations, as evaluated by flow cytometry (*n* = 4). The ratio with PI or annexin V positive cells were indicated as dead cells. **d**–**f** SH-SY5Y cells were exposed to the indicated concentrations of lidocaine (0, 1, 4, or 10 mM) for 24 h in the presence or absence of pretreatment with 10 μM recombinant human thioredoxin (rhTRX) for 2 h. Graphic depictions of (**d**) cell viability (*n* = 3), (**e**) caspase-3/7 activity (*n* = 3), and (**f**) cell death (*n* = 4), as determined by MTS, Apo-ONE™ Homogeneous Caspase-3/7, and flow cytometry analysis, respectively. All data were expressed as means ± standard deviations (SD). Differences between results were evaluated by two-way analysis of variance (ANOVA) followed by Dunnett’s test for multiple comparisons in each group. *p < 0.05 compared with the control cell population at time 0 h (no treatment). #*p* < 0.05 compared with the control treatment population in the same group

Next, we chose to examine the effects of TRX, an endogenous redox active protein, on lidocaine-induced cell death. First, however, we evaluated the effects of GGA, which is known to be an inducer of TRX [21, 22], on SH-SY5Y cells. Consistent with the results obtained using ROS scavengers, pre-treatment of SH-SY5Y cells with 5 μM and 10 μM GGA for 2 h resulted in reduced 4 mM lidocaine-induced cell death and caspase-3/7 activation (Fig. 4a–c). 10 μM GGA suppressed ROS generation elicited by 4 mM lidocaine (Additional file 3: Figure S3F). Even 10 μM GGA did not inhibit cell death or LDH release induced by 10 mM lidocaine (Fig. 4b and Additional file 3: Figure S3E). Surprisingly, however, treatment with 10 μg/ml rhTRX failed to suppress lidocaine-induced caspase-3/7 activation (Fig. 4d–f).

### Effects of synthetic antioxidants on mepivacaine and bupivacaine-induced cell death

**Fig. 5:**
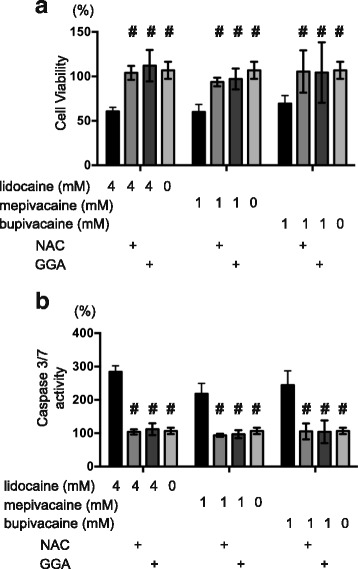
SH-SY5Y cells were exposed to the indicated concentrations of local anesthetics in the presence or absence of the indicated concentrations of 10 mM *N*-acetyl cysteine (NAC) and 10 μM geranylgeranylacetone (GGA) for 24 h. **a** Levels of cell viability and (**b**) caspase-3/7 activity were evaluated by MTS [3-(4,5-dimethylthiazol-2-yl)-5-(3-carboxymethoxyphenyl)-2-(4-sulfophenyl)-2H-tetrazolium] and Apo-ONE™ Homogeneous Caspase-3/7 Assay analyses (*n* = 3 for each). Data were expressed as means ± standard deviations (SD). Differences between results were evaluated by two-way analysis of variance (ANOVA) followed by Dunnett’s test for multiple comparisons. *p < 0.05 compared with the control cell population at time 0 h (no treatment). #*p* < 0.05 compared with the control treatment population in the same group

Lastly, we examined the in vitro effects of other local anesthetics on SH-SY5Y cells. Similar to those treated with 4 mM lidocaine, cells treated with 1 mM mepivacaine or 1 mM bupivacaine exhibited significantly reduced cell viability and increased caspase-3/7 activation (Fig. 5a and b), and these effects were blocked by treatment with 10 mM NAC or 10 μM GGA (Fig. 5a and b). These findings indicate that the anesthetic-mediated increases in cell death observed in this study are not exclusive to lidocaine.

## Discussion

In this study, we demonstrated that the local anesthetic lidocaine suppresses the mitochondrial ETC in neuronal SH-SY5Y cells in a dose- and time-dependent manner, thereby attenuating mitochondrial membrane potential, inducing ROS production, and activating caspase-9- and caspase-3/7-mediated apoptosis and necrosis. Moreover, we observed similar effects in cells treated with mepivacaine and bupivacaine. Intriguingly treatment with the antioxidants NAC and Trolox successfully suppressed these effects by scavenging the ROS derived from mitochondria.

### Lidocaine induces two types of cell death

Multiple studies have reported that clinically relevant concentrations (500 μM to 24 mM) of local anesthetics such as lidocaine are capable of inducing cell death in cells of neuronal origin as well as in established cell lines derived from cancerous tissues [23, 24]. Consistent with these findings, we demonstrated that 1 mM–10 mM lidocaine was sufficient to promote cell death in neuronal SH-SY5Y cells and HeLa cervical carcinoma cells (Figs. 1, 2, and Additional file 1: Figure S1).

### Lidocaine induces apoptosis and necrosis in a dose-dependent manner

There are at least two modes of cell death: apoptosis and necrosis [25]. Apoptosis is a strictly regulated (programmed) process involving the activation of specific cysteine proteases that is responsible for the ordered removal of superfluous, aged, or damaged cells. Notably, while this process plays critical roles in both health and disease, necrosis is solely the outcome of severe and acute injury. Apoptosis involves the regulated activity of catabolic enzymes (proteases and nucleases) within a near-to-intact plasma membrane, and is commonly accompanied by characteristic changes in nuclear morphology and chromatin biochemistry.

In this study, lidocaine treatment promoted caspase-3/7 and caspase-9 activation, as well as PARP cleavage, in SH-SY5Y cells (Fig. 1c and d). As such, these data indicate that lidocaine induces cell death via an authentic apoptosis pathway. Meanwhile, flow cytometry analyses demonstrated that treatment of cells with greater than 4 mM lidocaine especially 10 mM lidocaine also resulted in increased numbers of PI- and annexin V-positive cells (Additional file 1: Figure S1). These data strongly suggest that lidocaine elicits both types of cell death in a dose-dependent manner in vitro. Intriguingly, however, the observed increase in caspase 3/7 but not in cell death with LDH release, was significantly suppressed by treatment with NAC, Trolox and GGA. The evidence suggests that the antioxidants preferentially inhibit apoptosis rather than necrosis.

### Functional mitochondria are necessary for lidocaine-induced apoptosis but not for necrosis

Local anesthetics have been found to inhibit mitochondrial ETC at several points along the respiratory chain, and to inhibit F1-ATPase activity and adenine-nucleotide transport [26]. In this study, we also demonstrated the lidocaine inhibited oxygen consumption by mitochondria (Fig. 2c). Moreover, using ρ0 cells lacking mitochondrial DNA, we showed that functional mitochondria are required for lidocaine-induced cell death following caspase activation but not for lidocaine-induced necrosis with LDH release (Fig. 2b and e). These data strongly suggests that mitochondria comprise a critical target for lidocaine-induced cell death, particularly for apoptosis.

It was previously reported that dysfunction of the mitochondrial ETC due to mutations in OXPHOS subunits or treatment with chemical inhibitors is generally associated with increased production of mitochondrial ROS, including superoxide anions, hydroxyl radicals, and hydrogen peroxide [27]. Specifically, inhibitor studies using isolated mitochondria demonstrated that complexes I and III of the ETC can act as relevant sources of mitochondrial ROS [27]. Consistent with these findings, HEK293 cells treated with rotenone and antimycin A for inhibition of complexes I and III, respectively, exhibited increased ROS production and induction of oxidative stress [28]. Meanwhile, ROS production was not observed in ρ0 cells in this study (data not shown).

### ROS derived from mitochondria promote lidocaine-induced apoptosis

Another intriguing finding presented in this report was that both lidocaine-induced apoptosis and necrosis were ROS-dependent. The results presented in Fig. 3a clearly demonstrate that lidocaine treatment induced ROS generation. And while the precise origin of these ROS remains unclear, our findings strongly suggest that mitochondria play a critical role in this process (Fig. 2b and c). As such, these data imply that lidocaine-mediated cell death is dependent on mitochondria. Consistent with this conclusion, the mitochondrial DNA-deficient ρ0 cells were resistant to lidocaine treatment (Fig. 3b). Moreover, our data are consistent with a previous report demonstrating that tetracaine-induced apoptosis in rat cortical astrocytes is associated with increased ROS production [4].

### Effect of antioxidants on lidocaine-induced cell apoptosis

Oxidative stress in response to various external stimuli has been implicated in the induction of apoptosis. Specifically, oxygen free radicals induce DNA sequence changes and rearrangements that may trigger apoptotic cell death of neuronal cells. In this study, we provided the first evidence that ROS scavengers such as NAC and Trolox can significantly suppress lidocaine-induced cell death in vitro. Conversely, while treatment with rhTRX had no affect on apoptosis or necrosis, GGA, which is known to induce TRX expression and is itself an antioxidant, exerted a protective effect against lidocaine-induced cell death. Although the molecular mechanisms underlying these discrepancies are unclear, it is possible that extracellular administration of rhTRX is an ineffective method for modulating intracellular redox status [22, 29, 30]. Also, GGA has been shown to promote the expression of heat shock protein 70, which was reported to alleviate cellular stress and exert cytoprotective effects [31, 32]. As such, the observed beneficial effects of GGA on cell survival may be dependent on induction of HSP70 and not TRX. In any case, future studies using cells that overexpress TRX may better elucidate whether this protein can inhibit anesthetic-induced cell death.

### Limitations

There are several limitations to the present study. First, the established SH-SY5Y and HeLa cell lines were used exclusively for all experiments. Although SH-SY5Y cells are derived from neuronal tissue and exhibit several characteristics similar to neurons, our experimental results and conclusions cannot necessarily by extrapolated to neuronal injuries induced by local anesthetics in an in vivo setting. Furthermore, although we evaluated the effects of lidocaine on ρ0 cells, which lack mitochondrial DNA, the majority of the data were obtained using HeLa cells, primarily due to technical issues. Lastly, the results presented in this study demonstrate that local anesthetics negatively affect mitochondrial activity, thereby inducing apoptosis; however, the specific molecular targets have yet to be identified. As such, the identification of such targets comprises a critical goal for future studies.

## Conclusions

In this study, we demonstrated that the local anesthetics lidocaine, mepivacaine, and bupivacaine induce two types of cell death in neuronal cells in vitro. In particular, treatment with 1–4 mM lidocaine promoted apoptosis, while treatment with 10 mM lidocaine induced cell death with LDH release. Moreover, our data demonstrate that these compounds specifically target mitochondria, and that the ROS produced by mitochondria play an integral role in the observed induction of apoptosis. Lastly, we demonstrated that scavenging of ROS with antioxidants such as NAC, Trolox, and GGA preserved mitochondrial voltage and prevented apoptosis by suppressing caspase activation.

## Additional files

Additional file 1: Figure S1. Analysis of cell apoptosis by FACS. Levels of cell apoptosis were measured using an Annexin V-FITC Apoptosis Detection Kit (BioVision, Milpitas, CA, USA), according to the manufacturer’s instructions. For these analyses, SH-SY5Y cells were seeded into 6-well plates (3 × 10^5^ cells/well) and incubated overnight. The following day, cells were treated with the indicated concentrations of the appropriate drug(s) for varying lengths of time and harvested by centrifugation at 1200 rpm for 3 min. The culture supernatants were discharged, and the resulting pellets were resuspended in a mixture comprised of 500 μl binding buffer, 5 μl Annexing V-FITC, and 5 μl propidium iodide (PI; 50 μg/ml) for 5 min at room temperature in the dark and analyzed using a FACSCalibur flow cytometer (BD Biosciences, San Jose, CA, USA). (PDF 2009 kb)
Additional file 2: Figure S2. Mitochondrial membrane potential (ΔΨm). Mitochondrial membrane potential was determined by flow cytometry using a MitoPT™ JC-1 Assay Kit (ImmunoChemistry Technologies, Bloomington, MN, USA), according to the manufacturer’s instructions. For these analyses, SH-SY5Y cells were seeded into 6-well plates (3 × 10^5^ cells/well) and cultivated overnight. The following day, cells were treated with the indicated concentrations of the appropriate drug(s) for varying lengths of time and then pelleted by centrifugation at 1200 rpm for 3 min. Supernatants were discharged, and cells were resuspended in JC-1, incubated at 37 °C for 15 min in the dark, and collected by centrifugation at 1200 rpm for 3 min. Supernatants were again discharged and the remaining cell residues were suspended in 500 μl assay buffer. Samples were subsequently analyzed using a FACSCalibur flow cytometer (BD Biosciences, San Jose, CA, USA) equipped with CellQuest Pro™ software for the detection of red JC-1 aggregates (590 nm emission) or green JC-1 monomers (527 nm emission). (PDF 2041 kb)
Additional file 3: Figure S3. Results of HeLa cell-derivatives EB8 and HeEB1. (A) Oxygen consumption rate of HeLa cells, EB8 and HeEB1cells were demonstrated. (B) Graphic depiction of reactive oxygen species (ROS) production in HeLa cells and EB8 cells exposed to the indicated concentrations of lidocaine (0, 4, or 10 mM) for 6 h (*n* = 3). Data depict the ratio of ROS production in treated cells compared to that in the untreated control group (HeLa cells). (C) Activities of Caspase3/7 of HeLa cells and HeEB1 cells were demonstrated. (D) Levels of cell death were measured using an Annexin V-FITC Apoptosis Detection Kit evaluated by FACS were demonstrated. (E) Graphic depiction of the levels of cell death among treated and untreated cell populations. Cell death was evaluated by measuring the levels of lactate dehydrogenase (LDH) within culture supernatants (*n* = 3) in the presence or absence of 10 mM *N*-acetyl cysteine (NAC), 250 μM Trolox and 10 μM GGA. Control is LDH activity treated by lysis buffer. (F) Graphic depiction of reactive oxygen species (ROS) production in SH-SY5Y cells exposed to 4 mM) for 6 h (*n* = 3) in the presence or absence of 10 mM *N*-acetyl cysteine (NAC), 250 μM Trolox and 10 μM GGA. Data depict the ratio of ROS production in treated cells compared to that in the untreated control group. Data presented in A–E expressed as means ± standard deviations (SD). #*p* < 0.05 compared with the control cell population at the same time period. (PDF 587 kb)

## Abbreviations

NAC: *N*-acetyl cysteine
OCR: Oxygen consumption rate
ROS: Reactive oxygen species
TRX: Thioredoxin
