# Supplementary material for: The antioxidant N-acetyl cysteine suppresses lidocaine-induced intracellular reactive oxygen species production and cell death in neuronal SH-SY5Y cells
Source: BMC Anesthesiol. 2016 Oct 24;16:104. doi: 10.1186/s12871-016-0273-3 (PMC5078905; doi:10.1186/s12871-016-0273-3)
Supplement: Additional file 1: Figure S1. — Analysis of cell apoptosis by FACS. Levels of cell apoptosis were measured using an Annexin V-FITC Apoptosis Detection Kit (BioVision, Milpitas, CA, USA), according to the manufacturer’s instructions. For these analyses, SH-SY5Y cells were seeded into 6-well plates (3 × 10^5^ cells/well) and incubated overnight. The following day, cells were treated with the indicated concentrations of the appropriate drug(s) for varying lengths of time and harvested by centrifugation at 1200 rpm for 3 min. The culture supernatants were discharged, and the resulting pellets were resuspended in a mixture comprised of 500 μl binding buffer, 5 μl Annexing V-FITC, and 5 μl propidium iodide (PI; 50 μg/ml) for 5 min at room temperature in the dark and analyzed using a FACSCalibur flow cytometer (BD Biosciences, San Jose, CA, USA). (PDF 2009 kb) [file 12871_2016_273_MOESM1_ESM.pdf]

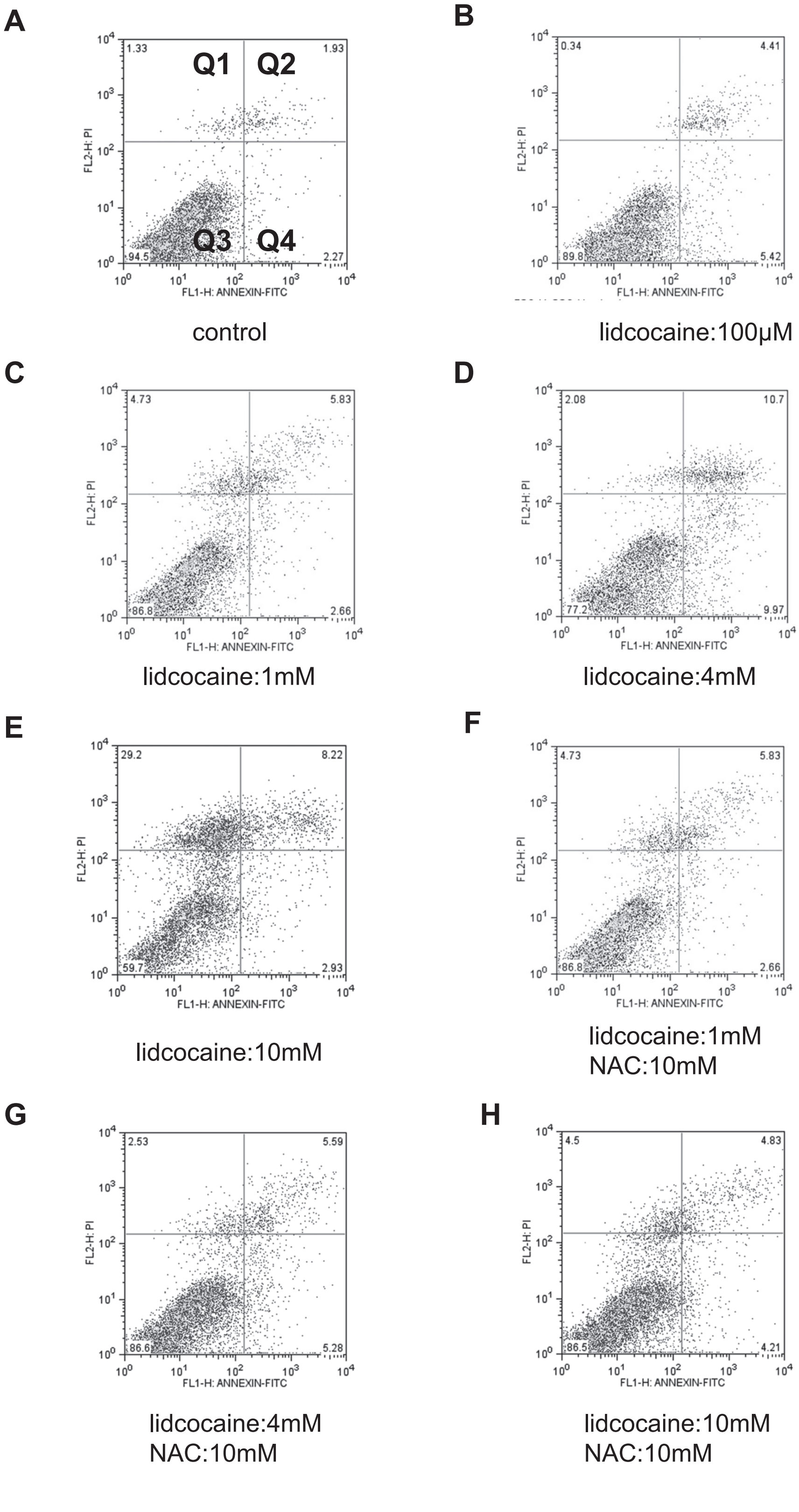

Okamoto Supplement S1
