# Supplementary figures and images for: The antioxidant N-acetyl cysteine suppresses lidocaine-induced intracellular reactive oxygen species production and cell death in neuronal SH-SY5Y cells
Source: BMC Anesthesiol. 2016 Oct 24;16:104. doi: 10.1186/s12871-016-0273-3 (PMC5078905; doi:10.1186/s12871-016-0273-3)

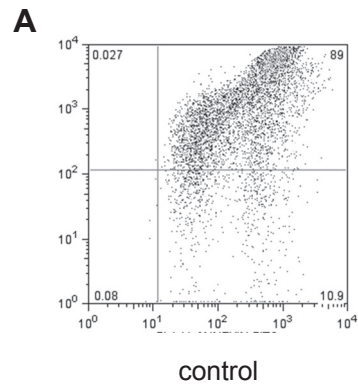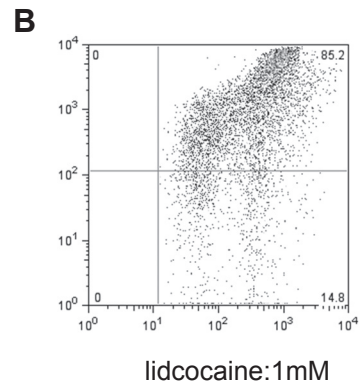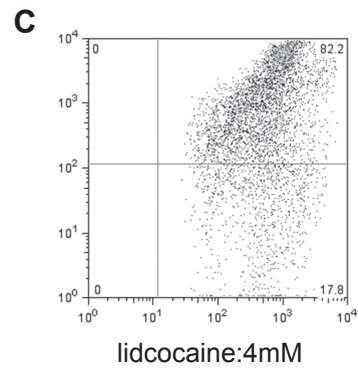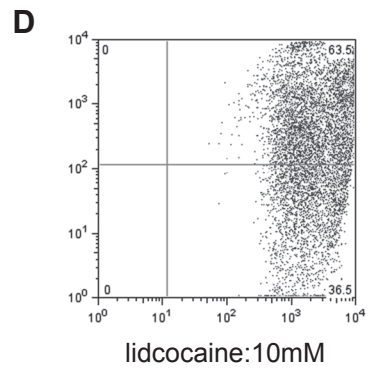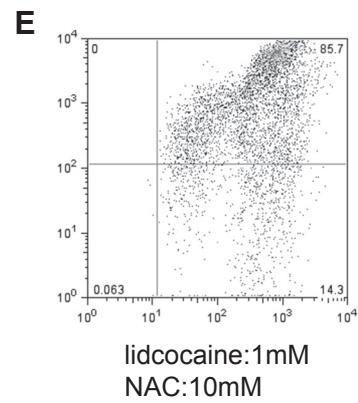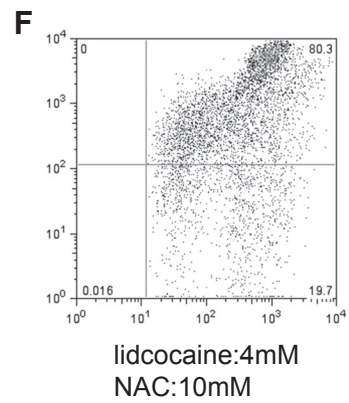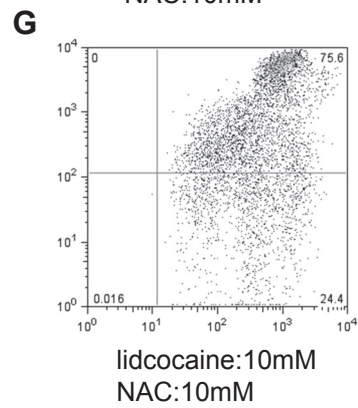

Supplement: Additional file 2: Figure S2. — Mitochondrial membrane potential (ΔΨm). Mitochondrial membrane potential was determined by flow cytometry using a MitoPT™ JC-1 Assay Kit (ImmunoChemistry Technologies, Bloomington, MN, USA), according to the manufacturer’s instructions. For these analyses, SH-SY5Y cells were seeded into 6-well plates (3 × 10^5^ cells/well) and cultivated overnight. The following day, cells were treated with the indicated concentrations of the appropriate drug(s) for varying lengths of time and then pelleted by centrifugation at 1200 rpm for 3 min. Supernatants were discharged, and cells were resuspended in JC-1, incubated at 37 °C for 15 min in the dark, and collected by centrifugation at 1200 rpm for 3 min. Supernatants were again discharged and the remaining cell residues were suspended in 500 μl assay buffer. Samples were subsequently analyzed using a FACSCalibur flow cytometer (BD Biosciences, San Jose, CA, USA) equipped with CellQuest Pro™ software for the detection of red JC-1 aggregates (590 nm emission) or green JC-1 monomers (527 nm emission). (PDF 2041 kb) [file 12871_2016_273_MOESM2_ESM.pdf]

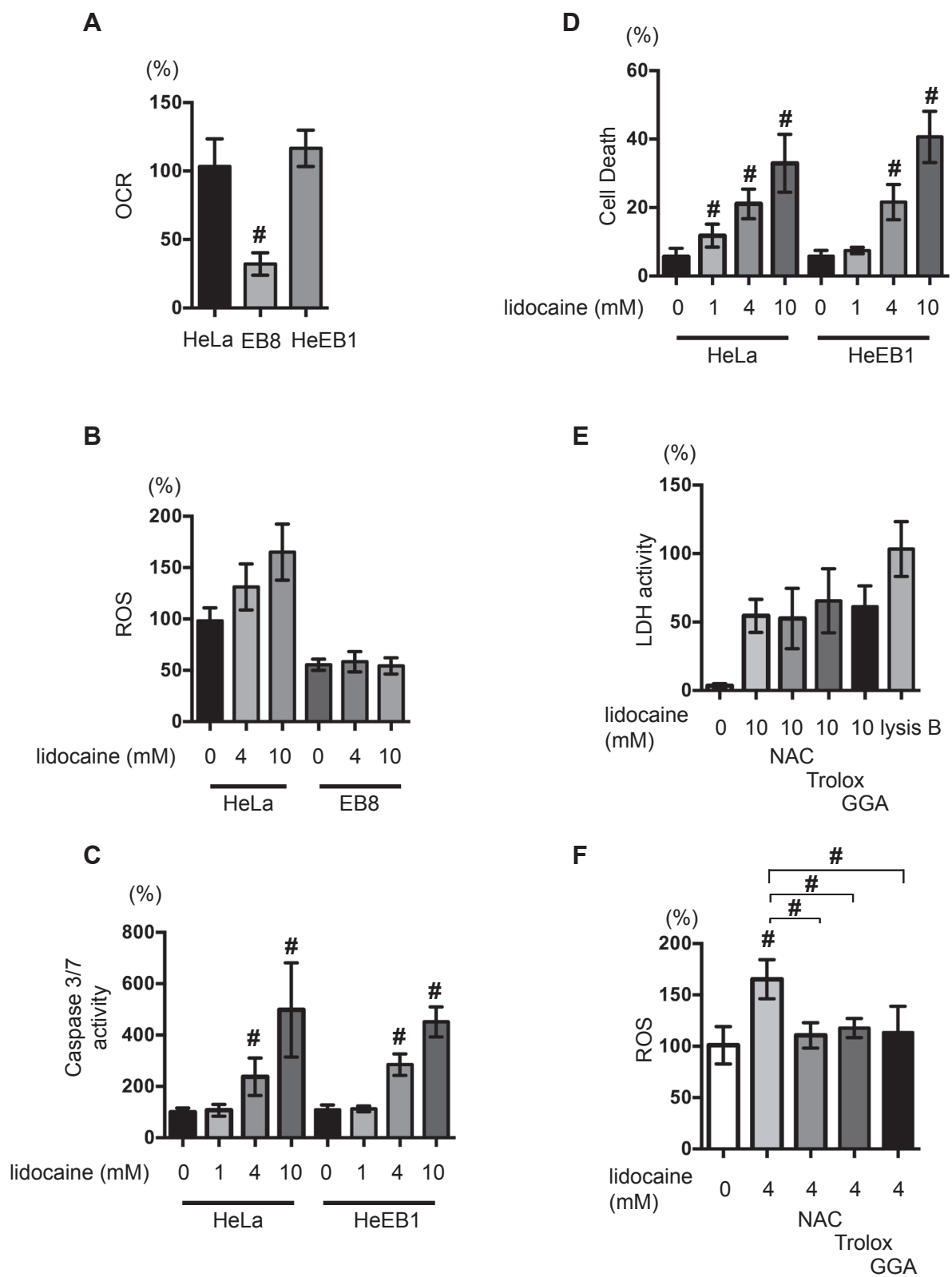

Supplement: Additional file 3: Figure S3. — Results of HeLa cell-derivatives EB8 and HeEB1. (A) Oxygen consumption rate of HeLa cells, EB8 and HeEB1cells were demonstrated. (B) Graphic depiction of reactive oxygen species (ROS) production in HeLa cells and EB8 cells exposed to the indicated concentrations of lidocaine (0, 4, or 10 mM) for 6 h (n = 3). Data depict the ratio of ROS production in treated cells compared to that in the untreated control group (HeLa cells). (C) Activities of Caspase3/7 of HeLa cells and HeEB1 cells were demonstrated. (D) Levels of cell death were measured using an Annexin V-FITC Apoptosis Detection Kit evaluated by FACS were demonstrated. (E) Graphic depiction of the levels of cell death among treated and untreated cell populations. Cell death was evaluated by measuring the levels of lactate dehydrogenase (LDH) within culture supernatants (n = 3) in the presence or absence of 10 mM N-acetyl cysteine (NAC), 250 μM Trolox and 10 μM GGA. Control is LDH activity treated by lysis buffer. (F) Graphic depiction of reactive oxygen species (ROS) production in SH-SY5Y cells exposed to 4 mM) for 6 h (n = 3) in the presence or absence of 10 mM N-acetyl cysteine (NAC), 250 μM Trolox and 10 μM GGA. Data depict the ratio of ROS production in treated cells compared to that in the untreated control group. Data presented in A–E expressed as means ± standard deviations (SD). #p < 0.05 compared with the control cell population at the same time period. (PDF 587 kb) [file 12871_2016_273_MOESM3_ESM.pdf]
